# The effect of irrigating solutions on the apical sealing ability of MTA Fillapex and Adseal root canal sealers

**DOI:** 10.15171/joddd.2016.040

**Published:** 2016-12-21

**Authors:** Richa Singh, Shankarappa Pushpa, Doraiswamy Arunagiri, Asheesh Sawhny, Abhinav Misra, Ramamurthy Sujatha

**Affiliations:** ^1^Senior Lecturer, Department of Conservative Dentistry and Endodontics, Rama Dental College, Hospital and Research Center, Kanpur, Uttar Pradesh, India; ^2^Professor and Head, Department of Conservative Dentistry and Endodontics, Rama Dental College, Hospital and Research Center, Kanpur, Uttar Pradesh, India; ^3^Principal, Professor and Head, Department of Conservative Dentistry and Endodontics, Maharana Pratap Dental College, Kanpur, Uttar Pradesh, India; ^4^Professor, Department of Conservative Dentistry and Endodontics, Rama Dental College, Hospital and Research Center, Kanpur, Uttar Pradesh, India; ^5^Reader, Department of Conservative Dentistry and Endodontics, Rama Dental College, Hospital and Research Center, Kanpur, Uttar Pradesh, India; ^6^Professor and Head, Department of Microbiology, Rama Medical College, Hospital and Research Center, Kanpur, Uttar Pradesh, India

**Keywords:** Microleakage, root canal irrigants, root canal preparation, root canal sealants, sealing efficacy, smear layer

## Abstract

***Background.*** Maximum sealing ability or adhesion of endodontic sealers can be achieved after effective removal of the smear layer. Endodontic irrigants assist in adequate removal of the smear layer, improving the retention mechanism. The aim of this study was to evaluate the effect of two different root canal irrigation solutions (5.25% NaOCl followed by 17% EDTA and QMix) on the apical sealing ability of two different root canal sealers (MTA Fillapex and Adseal).

***Methods.*** Forty-six single-canal teeth were divided into 4 experimental groups of 10 teeth each and a positive and negative group of 3 teeth each. The root canals were prepared using step-back technique. The teeth in groups 1 and 2 were irrigated with 5.25% NaOCl followed by 17% EDTA and the teeth in groups 3 and 4 were irrigated with QMix. Finally all the teeth were flushed with sterile saline and dried using paper points. Obturation was accomplished by gutta-percha using lateral condensation technique. MTA Fillapex sealer was used in groups 1 and 3 whereas Adseal was used in groups 2 and 4. Dye penetration method was used to evaluate apical leakage. Data were analyzed with Kruskal-Wallis and Mann-Whitney *U* tests using SPSS 14. Statistical significance was set at P < 0.05.

***Results.*** Group 3 showed maximum amount of apical leakage (3.7±0.3 mm) whereas group 2 exhibited the least amount of apical leakage (2.1 ± 0.4 mm) among all the experimental groups. Significant differences were found in the amount of apical leakage between all the groups (P = 0.00001).

***Conclusion.*** Within the limitations of this study, 5.25% sodium hypochlorite followed by 17% EDTA and Adseal resulted in the best apical seal.

## Introduction

The objective of root canal treatment is to prepare a clean, microbe- and debris-free canal for obturation.^1^ In addition to a proper root canal preparation and disinfection, an effective coronal and apical sealing ensures a long-term and successful endodontic treatment.^2^ A fluid tight seal for the entire root canal system is proposed as it prevents percolation of fluid from inflamed periapical tissues into inappropriately obturated canals.^1^ Though instruments are important in the removal of the infected dentin from the main canal, irrigants are crucial in areas inaccesible to instruments, such as lateral and accessory canals as well as fins and webs all over the canal.^3^Root canal irrigants serve multiple purposes during chemo-mechanical procedures as antimicrobial agents, dissolve organic tissue remnants, lubricate the dentinal walls and aid in flushing out debris, simultaneously assisting in the removal of the smear layer.^4^ Increased adhesion and sealing capacity is achieved after elimination of the smear layer.^5^

Sodium hypochlorite (NaOCl) is one of the most commonly used irrigants at a concentration ranging from 0.5% to 6%. It is well known for its antibacterial properties and tissue dissolving activity. Since, it has no effect on the inorganic part of the smear layer, Goldman in 1982 recommended that 17% ethylenediaminetetraacetic acid (EDTA) be used after NaOCl irrigation for complete removal of the smear layer.^3^

Haapasalo developed a new irrigant, QMix,^6^ which has substantial antimicrobial properties and is also effective in eliminating the smear layer.^7^It is a mixture of 2% chlorhexidine (a bisbiguanide, antimicrobial agent), 17% EDTA (a polyaminocarboxylic acid calcium-chelating agent) and cetrimide in distilled water with acceptable additional salt.^6^

Recently, a MTA-based sealer (MTA Fillapex) has been proposed as an endodontic sealer, which consists of MTA, salicylate resin, natural resin, bismuth oxide and silica. This material has attracted the researchers’ attention due to its excellent biocompatibility, bioactivity and osteoconductivity.^8^A study showed that this sealer has suitable physiochemical properties such as good radiopacity, flow and alkaline pH.^8^ Ferreira et al concluded that MTA-based sealers reduce the leakage into the root canal walls over time.^9^

Many characteristics such as adhesion to the tooth structure, long working time, ease of manipulation and good sealing ability of epoxy resin-based sealers made them the most commonly used sealers.^10^ A mixture of epoxy resins, amines, calcium phosphate, zirconium oxide, ethylene glycol salicylate, bismuth sub-carbonate and calcium oxide is incorporated into the recently introduced epoxy resin-based root canal sealer, Adseal.^11^ There are very few reports in the literature about its physical properties except for the reported radiopacity value.^10^

Various methods such as dye penetration, bacterial penetration, radio-labeled tracer penetration, dissolution of hard tissue, clearing of teeth, spectrometry of radioisotopes, electrochemical methods and gas chromatography have been used for evaluating the apical sealing properties of root canal sealers.^12,13^ According to Goldman et al^14^ (1989), the results of tracer penetration are hindered by the presence of entrapped air and are probably not very reliable.^14^ However, because of its sensitivity and amenity, the most common measurement used is dye penetration method.^12^ The longitudinal sectioning method facilitates examination of the exposed filling material and any dye penetration into the material at the interface of the dentinal wall on one side.^15^ The depth of dye penetration represents the gap between the root filling and canal walls.^12^

The aim of this study was to evaluate the effect of two different root canal irrigation solutions, i.e. 5.25% sodium hypochlorite followed by 17% EDTA and QMix on the apical sealing ability of two different root canal sealers, i.e. MTA Fillapex and Adseal.

## Methods

This study was approved by institutional Research Ethics Committee of Rama Dental College, Hospital and Research Center.

### Selection and preparation of samples

Forty-six single-rooted human mandibular premolar teeth extracted for orthodontic or periodontal reasons were selected for this study. Radiographs were taken to make sure of single canals and the teeth were examined under a stereomicroscope for any cracks, caries, external or internal resorption and canal calcifications. As for using extracted teeth, there were no special considerations from institutional Research Ethics Committee.

Before initiating the study, the coronal portion of the teeth were cut away near the cementoenamel junction using a diamond disc and a high-speed handpiece with water coolant, perpendicular to the long axis to achieve a length of 14 mm in all the samples. The teeth were then divided into four equal experimental groups (n=10). Three teeth were kept as positive and three as negative controls.

The procedure for preparation and obturation was performed by a single operator and all the groups were standardized.

The length of each canal was visually established by placing a #15 file (Dentsply-Maillefer, Ballaigues, Switzerland) into each tooth until the tip of the file was visible at the apex. The stopper was placed at cementoenamel junction which was the reference point. The working length was determined by subtracting 1 mm from the previous length. The canals were prepared up to #45 K-file using the step-back technique and the shaping of middle and coronal thirds was carried out by #3 and #4 Gates-Glidden drills (Dentsply-Maillefer, Switzerland).

After every change of endodontic file or Gates-Glidden drill, each canal was irrigated with 3 mL of irrigation solution. Two different endodontic irrigants were used. The teeth in groups 1 and 2 were irrigated with 5.25% NaOCl (Dentpro, Ammdent, Chandigarh, India) followed by 17% EDTA (Prevest DenPro, Jammu, India) and the teeth in groups 3 and 4 were irrigated with QMix (Dentsply, Tulsa Dental Specialties, Tulsa, OK). Both control groups (groups 5 and 6) were irrigated with sterile saline solution. Finally, the root canals were flushed with 2 mL of saline solution.

The apical patency was checked again by passing a #10 file through the apical foramen. Paper points were used to dry the root canals and standardized gutta-percha points (Dentsply-Maillefer, Switzerland) with proper tug-back at the working length were selected as master gutta-percha points. In groups 1 and 3 MTA Fillapex sealer (Angelus, Londrina, Parana) was used. Adseal sealer (Meta Biomed, Cheongju, South Korea) was used in groups 2 and 4. The obturation was completed with cold lateral condensation technique in all the experimental groups. The teeth were filled with gutta-percha without sealer in the positive control group and kept empty in the negative control group.

After obturation, the coronal access cavities of all the teeth were sealed with glass-ionomer cement (GC Fuji IX GP). The obturation of each tooth was assessed by radiographs. It was considered adequate if consistent gutta-percha without voids could be observed.

All the samples were stored in saline solution at 37°C for 48 hours to accomplish complete setting of the sealer. After 2 days, all the study specimens were thoroughly dried. The roots of all the experimental groups and the positive control group were coated with two coats of nail varnish except for the apical 2 mm. The entire root surface and apical foramen of the negative control specimens were completely covered with two coats of the nail varnish. The specimens were allowed to dry completely, then placed in 2% methylene blue dye solution (Central Drug House, New Delhi, India) and centrifuged at 3,000 rpm for 5 minutes. Afterwards the teeth were rinsed under tap water to remove the dye on external tooth surfaces.

### Leakage evaluation

The roots were longitudinally grooved with a diamond disc and then split into two halves with a chisel by levering with a plaster knife, making sure that the root canal filling was not penetrated. The dye penetration was measured from the apical to the coronal part of the root canal under a stereomicroscope (Leica Fluorescent Microscope, Wetzlar, Germany) with an ocular micrometer.

### Statistical analysis

SPSS 14 was used for analyzing the results. Kruskal-Wallis test was used to compare the mean dye penetration in the six groups. A P-value < 0.05 was considered to be significant. Pairwise comparisons of dye penetration in various groups were carried out by Mann-Whitney *U* test.

## Results

Apical leakage was observed in all the groups. The positive control specimens demonstrated maximum dye penetration of the root canal system; at the same time the negative control teeth exhibited the least dye penetration into the roots.

Table 1 shows means and standard deviations of various groups and comparison of mean scores by Kruskal-Wallis test. Pairwise comparisons of six groups were carried out with Mann-Whitney *U* test. Group 3 exhibited maximum amount of apical leakage (3.7 ± 0.3 mm) whereas group 2 showed the least amount of apical leakage (2.1 ± 0.4 mm) among all the experimental groups. The P-value for each group was < 0.001, which shows that there were highly significant differences between the groups.

**Table 1:** Comparison of six groups in relation to leakage values (mm) by Kruskal-Wallis test

**Groups**	**Mean**	**Standard Deviation**	**Median**	**Sum of ranks**	**H-value**	**P-value**
**Group 1**	2.9	0.3	2.9	264.50	54.2832	0.00001^*^
**Group 2**	2.1	0.4	2.0	162.00		
**Group 3**	3.7	0.3	3.8	435.50		
**Group 4**	3.4	0.3	3.4	362.00		
**Group 5**	4.7	0.5	5.1	551.00		
**Group 6**	0.3	0.1	0.3	55.00		

^*^P < 0.05 indicates significant differences between the groups.

**Figure 1.**
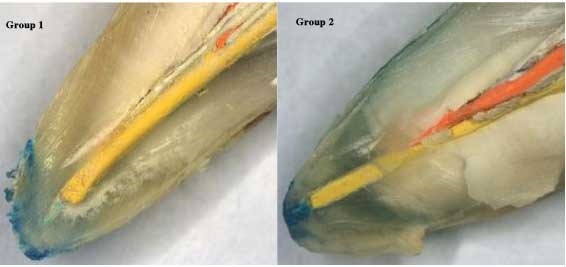
Apical region of teeth showing dye penetration in groups 1 and 2.

**Figure 2.**
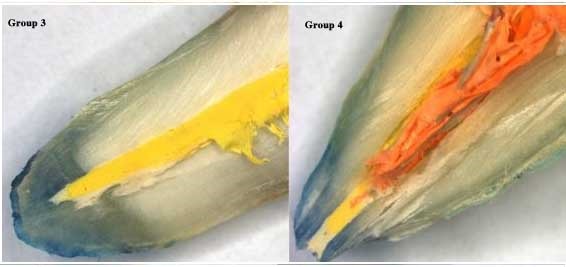
Apical region of teeth showing dye penetration in groups 3 and 4.

The apical sealing ability of sealers was affected by the irrigation solutions. Higher apical leakage values were observed with QMix as compared to 5.25% NaOCl, followed by 17% EDTA solution. However, the least amount of apical leakage was detected with 5.25% sodium hypochlorite, followed by 17% EDTA and Adseal combination. Figures 1,2and3 represent stereomicroscopic images of dye leakage in various groups.

**Figure 3.**
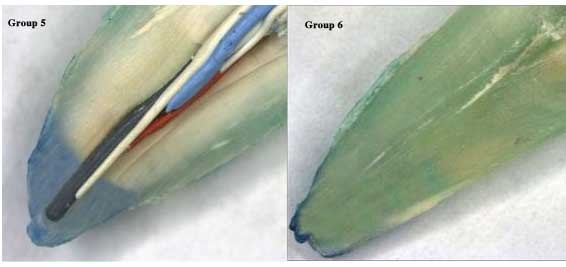
Apical region of teeth showing dye penetration in groups 5 and 6.

## Discussion

The study showed that the apical sealing ability of Adseal was better than that of MTA Fillapex when NaOCl was used followed by 17% EDTA as irrigation solutions. Also, the apical sealing ability of both Adseal and MTA Fillapex decreased when QMix was used as an irrigation solution.

One of the most common causes of endodontic therapy failure is apical leakage and is affected by many variables such as different obturation techniques, the physical and chemical properties of sealers and the presence or absence of the smear layer.^16^

The smear layer consists of an organic portion (coagulated proteins, necrotic and non-necrotic pulpal tissue, odontoblastic processes, saliva, blood cells, and microorganisms) and an inorganic portion (minerals from the dentinal structure).^5^ Elimination of the smear layer from the root canal system results in cleaner and unconcealed dentinal tubules, promoting a better apical sealing with the filling material by allowing easier infiltration of the dentinal tubules.^5^

NaOCl is the most commonly used irrigant; however, its action does not affect inorganic materials. EDTA complements the response of sodium hypochlorite by chelating calcium ions in dentin and making instrumentation of the root canal easier.^16^ QMix has been found to be effective in removing the smear layer and also has substantial antimicrobial properties.^7^ Dai et al (2011)^17^ and Stojicic et al (2012)^3^ reported that QMix was as effective as 17% EDTA in smear layer removal. This was based on the number of fully opened dentinal tubules.

Endodontic failure is mainly due to the residual microbial organisms remaining after chemo-mechanical preparation. Root canal filling ideally should entomb such residual microorganisms denying nutrient supply to them as well as preventing microbial access towards the periradicular tissues.^18^

In this study, dye penetration method was used to evaluate the apical sealing property of root canal obturating materials because of its sensitivity, simplicity and convenience. The depth of dye penetration apically or coronally represents the gap between the root filling and the canal walls.^12^

Hasheminia et al^19^ showed that the sealing ability of Adseal was comparable to that of AH Plus and other epoxy resin-based sealers.^19^ Studies by Sonmez et al^20^ and Ferreira et al^9^ showed that MTA Fillapex resulted in more microleakage than AH Plus and Topseal, respectively, when irrigating the canals with NaOCl and EDTA. The results of the current study were found to be consistent with these.

The better sealing ability of Adseal can be explained by the fact that epoxy resin sealer is considered contraction-free during setting, reactions which is responsible for its appropriate interfacial adaptation.^10^ Epoxy resins forms an intimate contact with dentin, remains micromechanically retained, reinforces the tooth structure and prevents re-contamination.^21^ Although it is believed that MTA Fillapex provides a good seal due to the expansion during setting, there is limited research about its physicochemical properties.^9^

MTA Fillapex showed more dye leakage (3.7±0.3 mm) than Adseal (3.4±0.3 mm) when QMix was used as an irrigation solution. The effect of QMix on apical sealing ability of root canal filling materials has not been reported yet; however, studies have shown that EDTA negatively interferes with the hydration of MTA.^22^

In the present study, dye leakage values were higher for groups irrigated with QMix (3.7±0.3 mm) than those irrigated with NaOCl and EDTA (2.9±0.3 mm) where MTA Fillapex was used as sealer. Similarly when Adseal was used as root canal sealer, dye leakage values for groups irrigated with QMix (3.4±0.3 mm) were higher than the groups irrigated with NaOCl and EDTA (2.1±0.4 mm).

This outcome might be attributed to the fact that QMix solutions cannot dissolve organic tissues in the root canal system. Hence, the remnants of these organic materials might interfere with the bonding of the sealer to the root canal walls when QMix is used as a root canal irrigant.^23^

Further long-term in vivo studies are required for the evaluation of sealing ability of sealers as well as the effect of root canal irrigants on the apical seal.

## Conclusion

Within the limitations of this ex vivo study it can be concluded that the apical sealing ability of both MTA Fillapex and Adseal decreased when QMix was used as an irrigation solution. NaOCl followed by 17% EDTA yielded better results than QMix. In addition, Adseal showed better sealing ability than MTA Fillapex. So to conclude, none of the groups showed complete fluid-tight seal.

## Acknowledgments

The authors have no one to acknowledge with regards to this work.

## Authors’ contributions

RS was responsible for the study design, data collection and compiling the manuscript. SP and DA were responsible for designing the research protocol and manuscript writing and reviewing. AS was responsible for collecting reviews for the study. AM was responsible for designing the methodology and performing the analysis and data compilation. RMS was responsible for reviewing and editing the manuscript. All the authors have read and approved the final manuscript.

## Funding

The study was self-funded.

## Competing interests

The authors declare no competing interests with regards to the authorship and/or publication of this article.

## Ethics approval

This study was approved by institutional Research Ethics Committee of Rama Dental College, Hospital and Research Center.
